# A putative “chemokine switch” that regulates systemic acute inflammation in humans

**DOI:** 10.1038/s41598-021-88936-8

**Published:** 2021-05-06

**Authors:** Nabil Azhar, Rami A. Namas, Khalid Almahmoud, Akram Zaaqoq, Othman A. Malak, Derek Barclay, Jinling Yin, Fayten El-Dehaibi, Andrew Abboud, Richard L. Simmons, Ruben Zamora, Timothy R. Billiar, Yoram Vodovotz

**Affiliations:** 1 Department of Surgery, University of Pittsburgh, W944 Starzl Biomedical Sciences Tower, 200 Lothrop St, Pittsburgh, PA 15213 USA; 2 Department of Computational and Systems Biology, University of Pittsburgh, Pittsburgh, PA 15213 USA; 3 Center for Inflammation and Regeneration Modeling, McGowan Institute for Regenerative Medicine, University of Pittsburgh, Pittsburgh, PA 15219 USA; 4 Center for Systems Immunology, University of Pittsburgh, Pittsburgh, PA 15213 USA

**Keywords:** Computational biology and bioinformatics, Systems biology

## Abstract

Systemic inflammation is complex and likely drives clinical outcomes in critical illness such as that which ensues following severe injury. We obtained time course data on multiple inflammatory mediators in the blood of blunt trauma patients. Using dynamic network analyses, we inferred a novel control architecture for systemic inflammation: a three-way switch comprising the chemokines MCP-1/CCL2, MIG/CXCL9, and IP-10/CXCL10. To test this hypothesis, we created a logical model comprising this putative architecture. This model predicted key qualitative features of systemic inflammation in patient sub-groups, as well as the different patterns of hospital discharge of moderately vs. severely injured patients. Thus, a rational transition from data to data-driven models to mechanistic models suggests a novel, chemokine-based mechanism for control of acute inflammation in humans and points to the potential utility of this workflow in defining novel features in other complex diseases.

## Introduction

Traumatic injury is a significant cause of morbidity and mortality and the leading cause of death in people under 55 years old^1,2^. In recent years, the outcomes landscape in blunt trauma has shifted from mortality to secondary complications such as multiple organ dysfunction syndrome (MODS) and nosocomial infection, leading to a prolonged length of stay (LOS) in the intensive care unit (ICU) and hospital^3^. This injury-induced critical illness has been attributed in large part to the inflammation and immune dysregulation elicited after trauma/hemorrhage^4–8^. Systemic acute inflammation is a complex process that occurs at multiple scales and involves the activation of signaling pathways that mobilize inflammatory cells and stimulate the systemic release of multiple inflammatory mediators such as damage-associated molecular pattern (DAMP) molecules, chemokines, and cytokines^9,10^. Numerous studies have investigated this response in the context of severe injury at the level of cellular mobilization^11^, genomic pathway activation^12^, and secreted mediators^13,14^. Analyzing circulating inflammatory mediators is particularly informative, as these are elevated following severe injury, and constitute the communication medium for organizing the response among the various cell types^9^.

Previous studies of systemic inflammation in trauma have either focused on association of dynamic patterns of inflammatory mediators with specific outcomes^13,15–20^ or building predictive mechanistic models from prior biological knowledge/literature^21^. Herein, we sought to identify early regulatory architectures directly from trajectories of circulating inflammatory mediators using dynamic network inference coupled with simulatable, quasi-mechanistic models.

## Results

### DyBN inference suggests a central, chemokine-based switching motif in trauma patients

**Figure 1:**
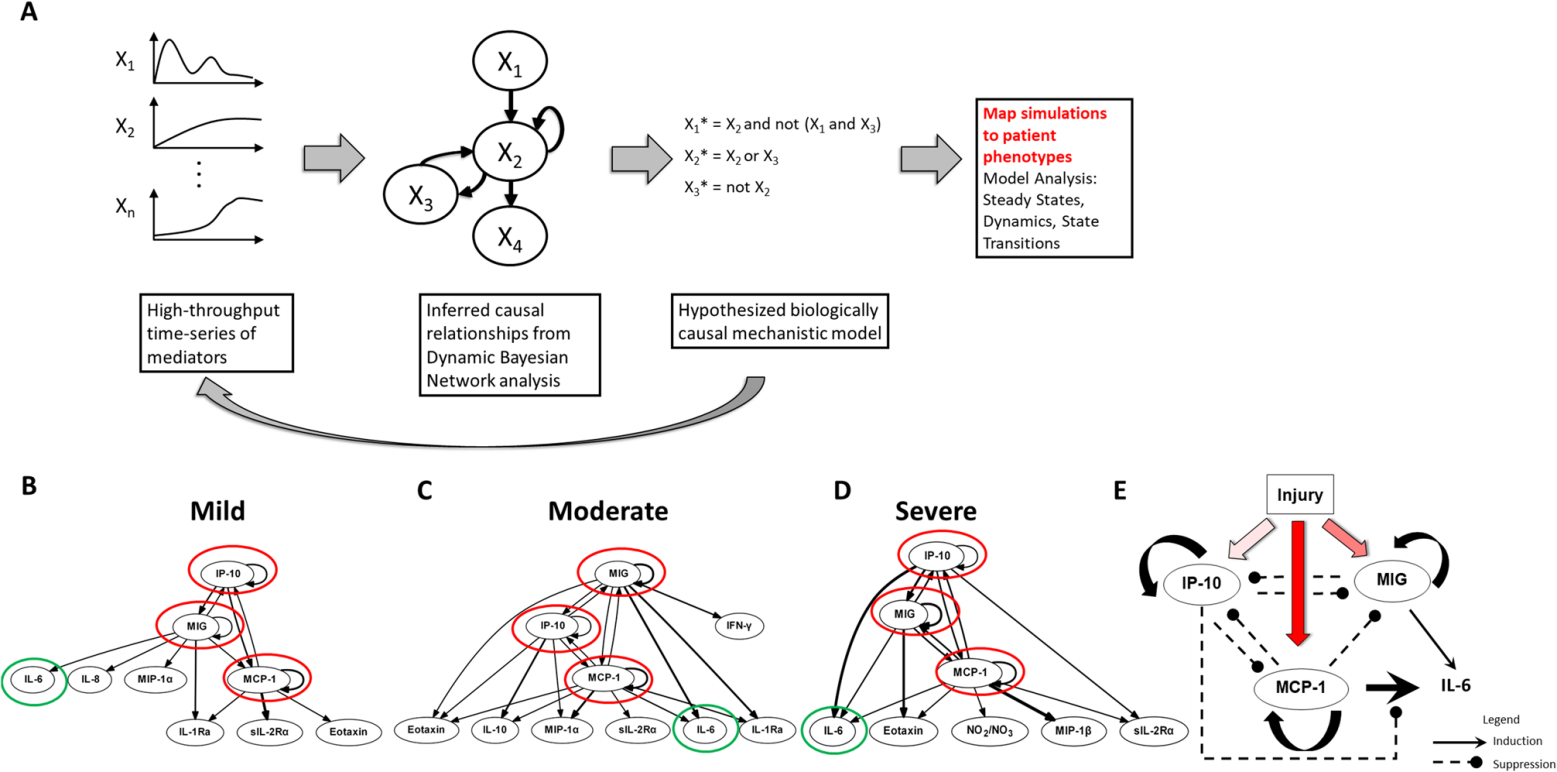
Workflow leading to a conceptual model of the “chemokine switch”. (Panel **A**): A schematic of the analysis workflow. Time courses of inflammatory mediators were measured in trauma patients and causal interactions inferred by DyBN analysis. The inferred network topology formed the basis a Boolean model which was simulated in silico. Results were compared to clinical trajectories to refine the model. (Panels **B-D**): DyBN consensus network structure for Mild, Moderate and Severe Injury. Panel **E**: Boolean model structure with cross-regulation among chemokines MIG, IP-10, and MCP-1.

We sought initially to identify potential feedback architectures in the systemic inflammatory responses of trauma patients as a function of injury severity. Accordingly, we focused on three groups of trauma patients matched for age and gender distribution but differing in injury severity, and with progressively worse clinical outcomes as a function of injury severity^20,21^ (Table 1). The initial step in our workflow (Fig. 1A) involved obtaining a time-series dataset of circulating, protein-level inflammatory mediators. Time courses of 24 systemic inflammatory mediators from each patient were used as input for the DyBN inference algorithm. This analysis suggested a consistent core motif across all three injury severity groups, involving the chemokines MCP-1, MIG, and IP-10 with cross-regulation among them and with the cytokine IL-6 as a shared output node (Fig. 1B-D)^20^. Notably, IL-6 levels generally correlate with injury severity and adverse outcomes such organ dysfunction, and therefore serve as an excellent marker for the magnitude of the systemic inflammatory response and risk for organ dysfunction^9,22^. We note that several inflammatory mediators were significantly different by ANOVA between the moderate and severe injury patient sub-groups (Figure S1) but were not identified as nodes in the DyBN networks, likely due to the major methodological differences between ANOVA and DyBN. These mediators may represent a consequence of the chemokine-based feedback structure, similar to IL-6. Thus, data-driven modeling could discern potential proximal feedback structures in an extensive time course of inflammatory mediators from injured patients, which in turn are associated with a key biomarker of morbidity and mortality following traumatic injury.

**Table 1:** Demographics, clinical outcomes, and co-morbidities of mild, moderate, and severely injured patients. Cohorts were age- and gender-matched. Length of stay in the ICU (ICU LOS), total length of stay (Total LOS), and days on mechanical ventilation increase with injury severity. Values are expressed as median (1st-3rd quartile range).

	Mild (n = 48)	Moderate (n = 47)	Severe (n = 47)	P value
ISS*	10 (9–13)	20 (17–22)	29 (27–35.5)	< 0.001
**Demographics**				
Age	42.5 (31.5–51)	41 (25.5–51.5)	43 (26.5–52)	0.71
Gender	M = 33 F = 15	M = 33 F = 14	M = 32 F = 15	0.97
**Clinical Outcomes**				
ICU LOS*	2 (2–4)	4 (2–7)	9 (4–13)	< 0.001
Total LOS*	6.5 (3.75–12)	9 (5–15)	14 (9–24)	< 0.001
Mechanical Ventilation*	0 (0–1.25)	1 (0–2)	4 (1–10)	0.0002
**Co-morbidities**				
Asthma, n (%)	3 (6.2%)	2 (4.2%)	2 (4.2%)	0.87
COPD, n(%)	2 (4.2%)	1 (2.1%)	0	0.78
Diabetes Mellitus, n (%)	2 (4.2%)	5 (10.6%)	4 (8.5%)	0.48
Hypertension, n (%)	8 (16.7%)	9 (19.1%)	11 (23.4%)	0.71
Psychiatric illness, n (%)	8 (16.7%)	4 (8.5%)	5 (10.6%)	0.44
Thyroid disease, n (%)	6 (12.5%)	2 (4.2%)	1 (2.1%)	0.89
Alcohol intake, n (%)	5 (10.4%)	3 (6.4%)	5 (10.6%)	0.72
Smoker, n (%)	6 (12.5%)	4 (8.5%)	3 (6.4%)	0.57
Other, n (%)	14 (19.2%)	16 (34%)	18 (38.3%)	0.64
None, n (%)	19 (39.6%)	20 (42.5%)	19 (40.4%)	0.95

### Logical model based on DyBN results captures population behavior

DyBN inference suggested the possibility of a novel feedback architecture based on correlations among the multitude of inflammatory mediators assessed. However, correlation is not causality. To test whether MCP-1, MIG, and IP-10 affected their own levels, the levels of the other two chemokines, or the levels of IL-6 in a manner that depended on injury severity, we constructed a logical model that posits the hypothesis schematically represented in Fig. 1E. Because the parameters are integrated during the process of learning the DyBN structure, we can only translate the directionality of the interactions but not whether the interactions are positive or negative (representing activation or inhibition). Thus, when constructing the logical model, we initially ascribed positive vs. negative interactions based on plausible mechanisms from the literature and our own hypotheses. We then adjusted the labels and iterated through several versions of the model in order to arrive at the final model that best recapitulated the observed trajectories of MIG, MCP-1, IP-10, and IL-6. A similar approach was employed to deduce the logical rules governing combinations of interactions (AND vs OR). The model that best reproduced the observed population behavior of moderately and severely injured patients postulated that each chemokine upregulates its own expression while downregulating the expression of the other two in a manner dependent on injury severity, a hypothesis supported by studies regarding the negative cross-regulation of chemokines by other chemokines (generally at the level of shared receptors [e.g. CXCR3, which is shared by MIG and IP-10])^23–28^.The set of rules for this initial logical model is provided in Supplemental Table S1. The interactions of the model, which form a series of hypotheses, can be described as follows:

- Moderate injury induces IP-10 and MIG, while severe injury induces these as well as MCP-1 (in line with prior studies in both mouse^29^ human blunt trauma^16,18,30^).
- IP-10 has positive feedback on itself^31,32^, and both MIG and MCP-1 must be active (have a nonzero value) to suppress IP-10. Furthermore, the suppression of IP-10 is an "AND" interaction, and thus both MIG and MCP-1 must be non-zero in order for IP-10 to be fully suppressed.
- MCP-1 can be induced either by severe injury alone, or by positive feedback on itself (in line with the positive feedback described in monocytes^33^), but high IP-10 levels suppress this self-feedback. MCP-1 can reach high levels only in the combination of severe injury, moderate MCP-1, and lack of high IP-10, i.e. self-feedback is not sufficient to reach high MCP-1 levels. Thus, the model rules clearly dictate that MCP-1 can only reach high levels in severe injury. Also, as long as injury is severe, MCP-1 will remain at least at moderate levels even with high IP-10 (Supplemental Figure S2A).
- MIG has self-feedback^32^ but is suppressed when both IP-10 is high and MCP-1 is active.
- IL-6 is activated by both MIG and MCP-1. However, high levels of IL-6 are induced only when MCP-1 is present (in line with our prior studies showing reduced IL-6 production by hepatocytes from MCP-1-null mice^16^) and suppressed by high IP-10. In an alternative formulation of this rule, MIG reduces the degree to which IL-6 is induced by MCP-1, resulting in essentially identical predicted IL-6 dynamics (data not shown).

The dynamics of the model are as follows. When all elements are initialized to zero, they remain at zero (Supplemental Figure S2B). When injury is set to “moderate,” IP-10 rises to its highest level while all other variables remain at zero (Supplemental Figure S2C). When injury is set to “severe,” IP-10 rises to its highest level. Also, MCP-1 and then IL-6 rise to their highest levels before gradually reaching steady state at a moderate level (Supplemental Figure S2D).

### Model verification against data from trauma patients

**Figure 2:**
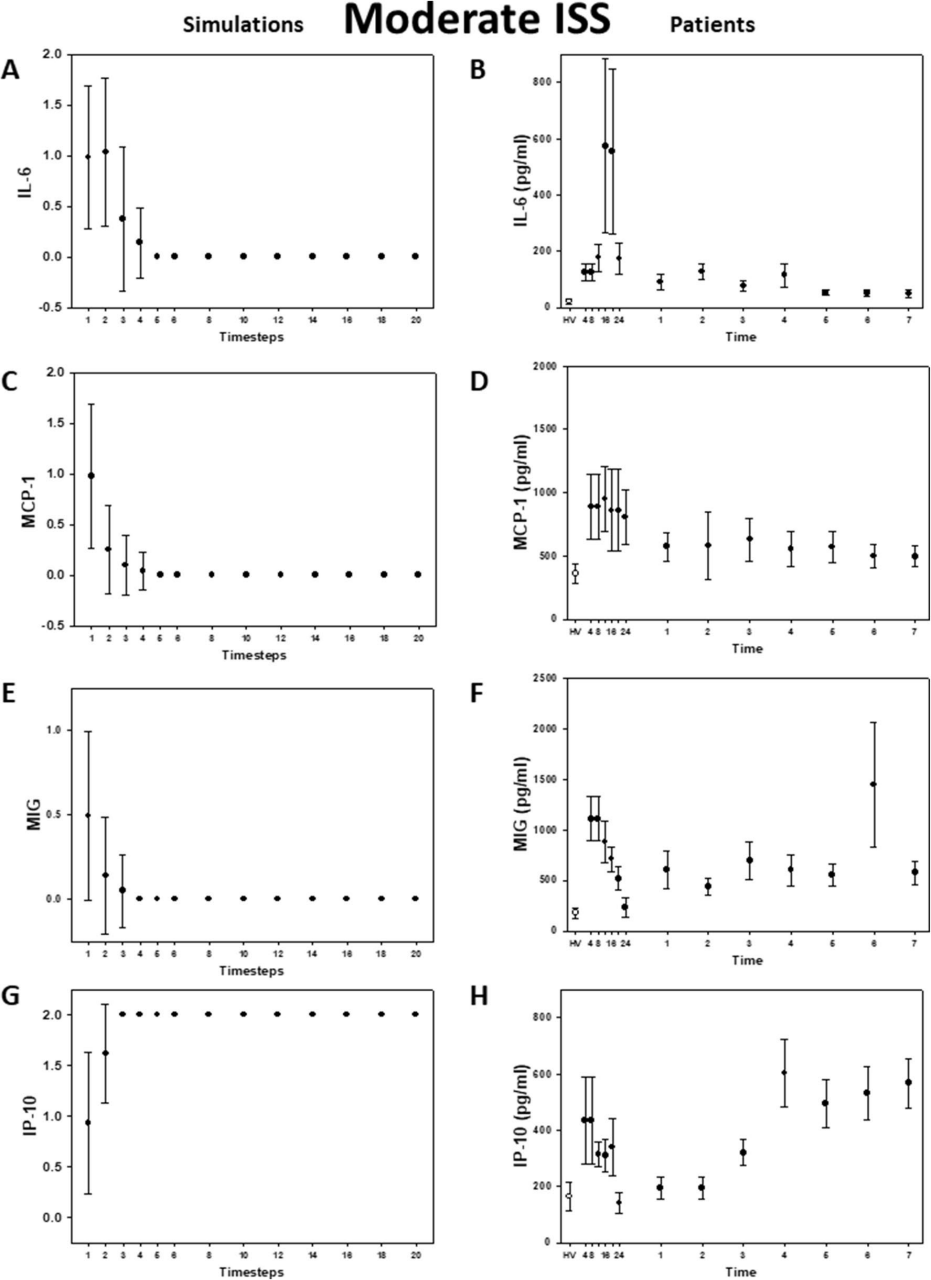
Inflammatory mediator trajectories for Moderate Injury: Simulations vs. data from trauma patients. Left column: 500 simulations were run with random initial conditions. Plot shows mean plus standard error for each time step. Right column: Patient data shown as mean with standard error for each time point. Healthy volunteer (white circles) included for reference.

**Figure 3:**
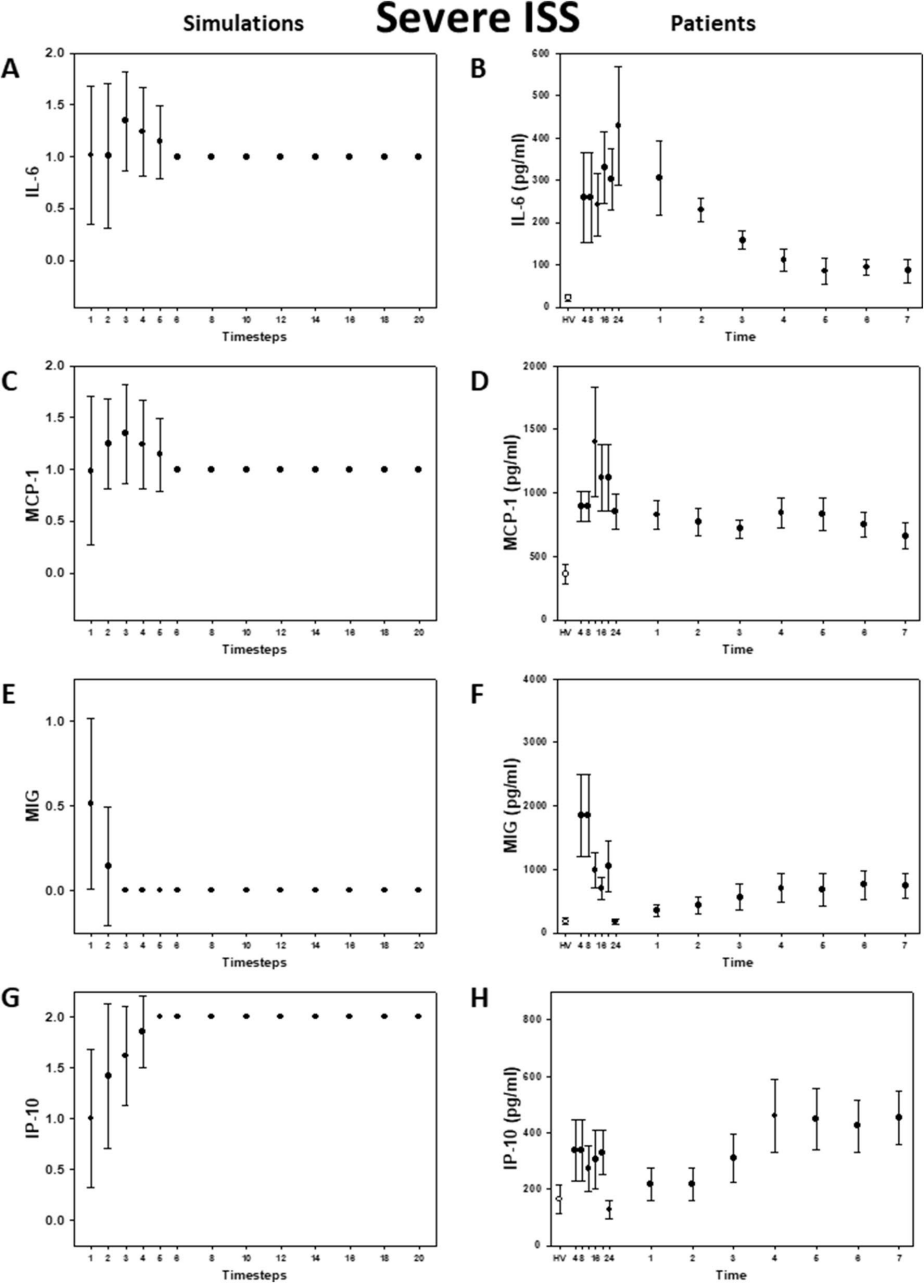
Inflammatory mediator trajectories for Severe Injury: Simulations vs. data from trauma patients. Left column: 500 simulations were run with random initial conditions. Plot shows mean plus standard error for each time step. Right column: Patient data shown as mean with standard error for each time point. Healthy volunteer (white circles) included for reference.

To mimic a population of patients starting with random baseline values of each of the inflammatory mediators, the model was next simulated with random initial conditions. Our simulations matched qualitatively with the clinical data, and, importantly, were able to capture the key differences in the dynamics of MCP-1 and IL-6 in moderately and severely injured patients (Fig. 2A-B vs 3A-B). In the simulations, IL-6 reached a steady state at low levels within the first 5 time-steps for moderate injury (Fig. 2A), whereas this cytokine remained at higher levels for longer and reached steady state at a moderate (non-baseline) level in the severe injury case (Fig. 3A). In agreement with these simulations, IL-6 levels returned to near baseline values within the first 24 h in moderately injured patients (Fig. 2B), whereas IL-6 levels remained higher for up to three days in severely injured patients (Fig. 3B). The simulations for MCP-1 (Fig. 2C and 3C) showed the same behavior as in the patients (Fig. 2D and 3D). MIG and IP-10 trajectories in both simulations (Fig. 2E, 2G, 3E, and 3G) and patients (Fig. 2F, 2H, 3F, and 3H) did not differ qualitatively between moderate and severe injury, respectively.

### Model refinement suggests an intermediate step delaying IP-10 induction following injury

**Figure 4:**
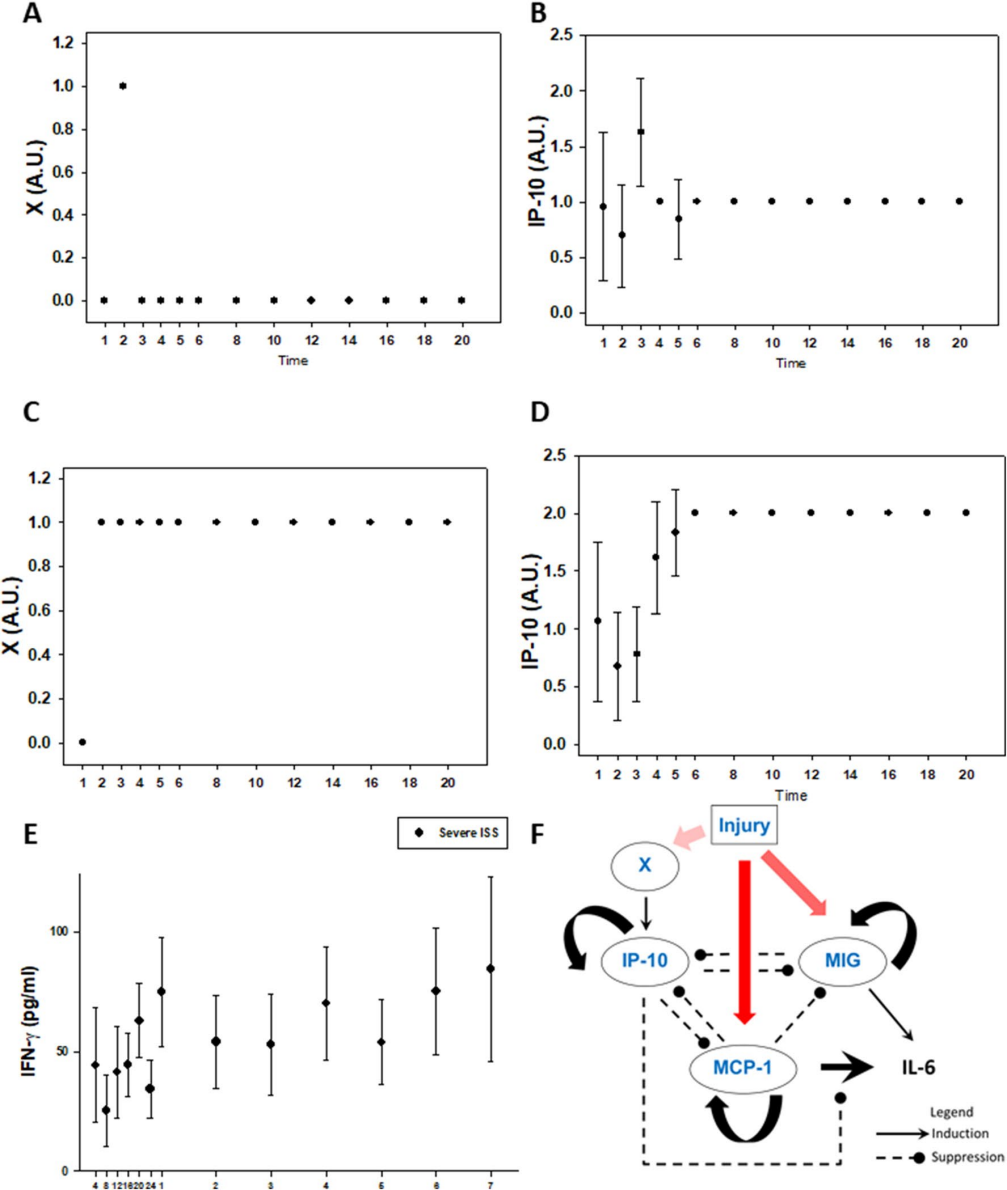
Addition of a putative node improves IP-10 simulations. A new variable, *X*, was added upstream of IP-10 to introduce a delay due to the observed slow rise in patient IP-10 trajectory. A spiky trajectory for *X* (Panel **A**) produces an IP-10 trajectory (Panel **B**) that is inconsistent with patient data. A step rise for *X* (Panel **C**) produces an IP-10 trajectory (Panel **D**) that matches well with patient data. Coincidentally, out of the measured inflammatory mediator profiles, the trajectory of circulating IFN- γ most closely resembles a step increase trajectory (Panel **E**). (Panel **F**): Model schematic with addition of new variable *X*.

Despite the overall qualitative agreement between simulations and data, the simulations for IP-10 showed a monotonic rise to a steady state high level (Fig. 2G and 3G), whereas in the patients there was an early dip and delayed rise (Fig. 2H and 3H). In order to address this discrepancy, we hypothesized an additional node (labeled *X*), positioned upstream of IP-10, which could delay IP-10 induction following injury. We examined both spiky (Fig. 4A) and sustained (Fig. 4B) dynamics of node *X* and observed that the IP-10 trajectory corresponding to the latter behavior most closely fit the data in our patient cohort (compare simulations in Fig. 4D vs. data in Fig. 3H). We compared the predicted trajectory for node *X* (Fig. 4C) to all the inflammatory mediators measured in the patient cohort (see Supplemental Figure S1) and found a reasonable qualitative match between the dynamics of node *X* and those of circulating IFN-γ (Fig. 4E). Notably, IFN-γ is the key cytokine that induces IP-10 (hence IP-10′s name, “IFN-γ-inducible protein of 10 kDa)^34^. Thus, a mechanistic computational model was used to impute a plausible, novel node not inferred directly in the underlying data in a manner consistent with known biology and observed data in humans (Fig. 4F).

### Model validation based on a patient sub-population with low initial MCP-1 levels

**Figure 5:**
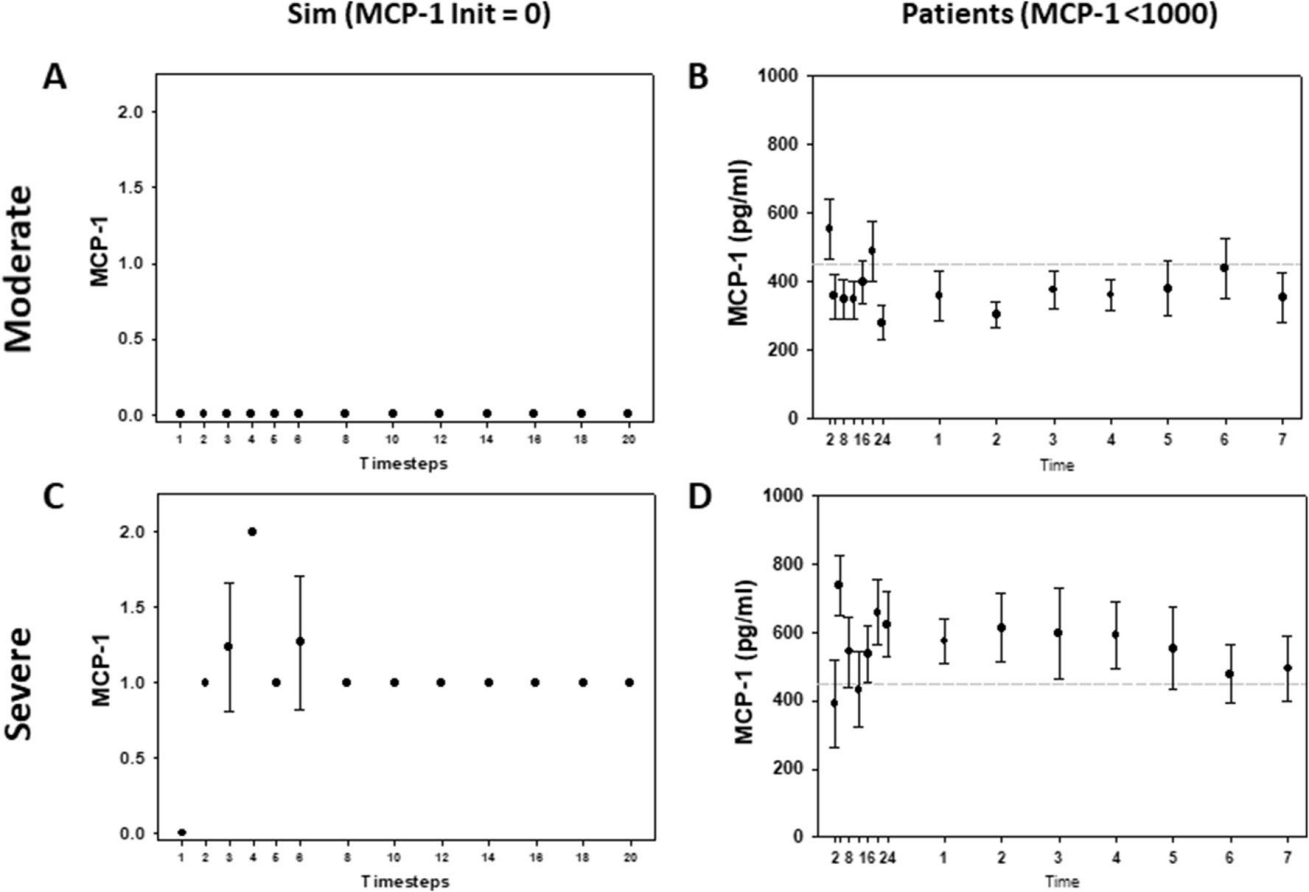
Logical model captures differences in Moderate vs. Severe Injury patients with low MCP-1. Simulation with moderate injury and low initial MCP-1 (Panel **A**) shows MCP-1 remaining low, whereas simulation with severe injury and low initial MCP-1 (Panel **C**) shows a rise and higher sustained MCP-1 levels. Similarly, patients with low initial MCP-1 and moderate injury (Panel **B**) exhibit lower MCP-1 levels throughout time course as compared to patients with severe injury (Panel **D**). Data in (Panels **B** vs. **D**) are significantly different (P < 0.05) by Two-Way ANOVA.

We next sought to validate our refined logical model. Predictions of all mechanistic computational models are, to some extent, dependent on initial conditions^35^. Accordingly, we tested whether specific initial conditions of one of the elements in our model combined with injury severity resulted in model predictions that matched clinically observed cytokine trajectories in trauma patient sub-populations that exhibited those attributes. As the model was calibrated to the behavior of the overall trauma patient population using random initial conditions, we consider this analysis a form of validation. A previous study from our group had shown that the outcomes of trauma patients could be segregated based on circulating MCP-1 levels^16^. Therefore, we chose the cutoff levels of circulating MCP-1 used in that study: levels lower than 1000 pg/ml were associated with patients whose clinical outcomes were better than those of patients with circulating levels greater than 1500 pg/ml^16,36^. Accordingly, we set 1000 pg/ml MCP-1 to correspond to an initial condition of low MCP-1. We compared the responses of patients with low MCP-1 under moderate or severe injury and observed that MCP-1 levels were significantly higher in patients with severe injury compared to patients with moderate injury (P < 0.05, 2-way ANOVA, Fig. 5B vs 5D). Correspondingly, model simulations showed that following moderate injury, MCP-1 levels that started at a low level remained at a lower level. In contrast, MCP-1 levels were predicted to rise following severe injury when starting with the same low initial condition of MCP-1, and this behavior was observed in the corresponding patient sub-population (Fig. 5A vs Fig. 5C). Thus, a quasi-mechanistic model constructed and refined following an initial data-driven model could reproduce key behaviors on which it was not trained explicitly, suggesting a novel interaction between initial levels of MCP-1 and the subsequent graded response to injury.

### State transition analysis reveals differences in the evolution of trauma-induced systemic inflammation

**Figure 6:**
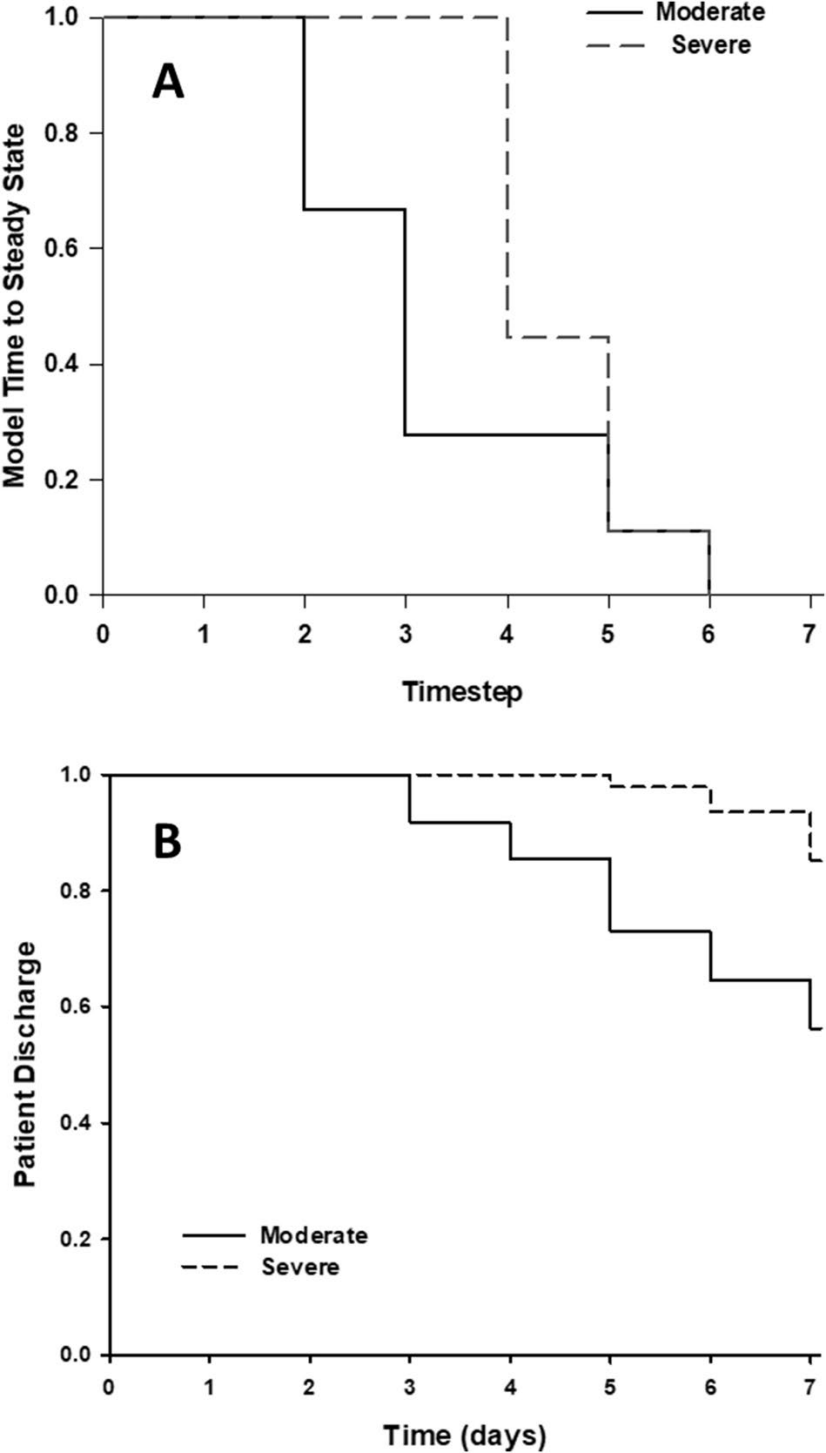
Logical model captures differences in patient discharge in Moderate vs Severe Injury patients. Kaplan–Meier style survival curve with endpoint as steady state (simulations, Panel **A**) or patient discharge (patients, Panel **B**). Simulations of moderate injury reach steady state sooner than severe injury (top panel). Similarly, patients with moderate injury are discharged sooner than severely injured patients (bottom panel).

Finally, we hypothesized that we could glean additional insights about post-injury inflammation and the role of the putative “chemokine switch” by examining the state transition diagram of our logical model. Although there was only one steady state for each injury severity irrespective of initial conditions, we investigated whether the time to reach that steady state varied, by examining the state transition graphs for the logical model (Figure S3). Our simulations are deterministic, so there is only one path from each initial state to the final steady state. For severe injury, overall, the simulations take longer to reach steady state than for moderate injury (Fig. 6A). Given that inflammation and associated injury-induced persistent critical illness are considered key determinant of a given trauma patient’s length of stay in the hospital^4–9^, we hypothesized that this inflammatory state transition would correlate with hospital discharge rates (Fig. 6A vs B). We observed that the model was indeed able to capture the earlier hospital discharge of moderately vs. severely injured patients.

## Discussion

In the present study, we inferred initial network connectivity from time-courses of circulating inflammatory mediators in human trauma patients to define central nodes and potential regulatory architectures. This dynamic network analysis suggested a core set of interactions among the chemokines MCP-1/CCL2, MIG/CXCL9, and IP-10/CXCL10 upstream of the cytokine IL-6. Building on the inferred network with biological mechanism and hypotheses, we constructed a Boolean model of this “chemokine switch” motif that we inferred from the network analysis. Predictions from this Boolean model matched data from trauma patients, identified a missing node, and correlated to differences in LOS between moderate and severe injury groups.

We have suggested previously that a workflow involving extensive time courses of data, data-driven modeling (e.g. network inference), and mechanistic modeling could serve to decipher the complexity of acute inflammation and other complex biological processes^9,37,38^. Herein, we demonstrate one example of this workflow, in which we utilized extensive time course data on systemic inflammatory mediators, suggested a potential regulatory architecture, tested this hypothesis computationally using a Boolean framework, inferred missing network nodes, and validated aspects of this model using separate data sets obtained from injured patients. Boolean models – with their state-based structure and qualitative underpinnings – represent a natural partner to data-driven network models. In contrast to reaction network models that are most often constructed as systems of differential equations, logical models do not require quantitative parameters. In this approach, the network elements are represented with discrete variables, and interactions are defined by logical rules. The resulting model allows exploration of dynamical and steady state behavior while providing qualitative comparison to experimental data.

Previous studies have shown that chemokines can be activated either directly by injury or indirectly through cytokines such as IFN-γ^39,40^. Our results in the present study support a highly nuanced role for chemokines in the setting of systemic inflammation induced by traumatic injury, as opposed to a more common view that chemokines are largely redundant in their pro-inflammatory effects. The regulatory architecture inferred computationally in this study – and documented in various related manifestations in other dynamic network studies in human blunt trauma^19,41,42^ as well as pediatric liver failure^43^ – is plausible based on numerous prior studies. For example, the switching architecture could be based at least in part on shared receptors, since CXCR3 is the receptor for both IP-10 and MIG^23–28^. Notably, previous studies have shown that MCP-1 ^16,18^, MIG ^30^, and IP-10 ^41^ are all potential biomarkers of adverse outcomes in human blunt trauma patients. Furthermore, recent studies in COVID-19 have suggested IP-10 as a major feature of this form of critical illness^44^ as well as being a biomarker of multiple other infectious diseases^45^. Our model suggests a role for IP-10 in moderating more severe inflammation, a hypothesis supported by the protective role of IP-10 in the context of SARS^46^.

Our modeling studies are based on levels of circulating inflammatory mediators, which may be derived from circulating cells or from one or more tissues/organs; recent modeling work from our group has pointed to a complex, cross-organ spatiotemporal cascade of acute inflammation that eventually leads to systemic spillover in endotoxemic mice^47^. Those studies did not point to specific cellular sources, however. While an indirect measure of cellular populations, chemokines might reflect the activities of pro- vs. anti-inflammatory neutrophils^48–50^ and eosinophils^51^; it is unclear if this occurs in other leukocytes as well. Ultimately, a multiscale model is required to connect changes in systemic inflammatory mediators to the underlying tissue-, cell-, and molecular/signaling-scale mechanisms in a complex setting such as traumatic injury^52^. As a rational approach toward such a model, we utilized network inference of abstracted interactions among cytokines to generate hypotheses about mechanism that can be further tested and eventually incorporated into a multiscale, mechanistic model. Since most evidence suggests that either insufficient^53^ or self-sustaining^54^ inflammation drives the pathobiology of trauma/hemorrhage and subsequent processes such as nosocomial infection-induced sepsis, we hypothesize that the putative “chemokine switch” motif that we have identified through computational modeling is an important regulator of these inflammatory regimes^9^. Thus, acute inflammation due to traumatic injury represents a highly complex and coordinated response which can become dysregulated when thresholds of injury are exceeded^9,10^. Factors other than severity are also likely to determine the nature of the proximal mediator structures. These include patient-specific factors such as gene polymorphisms^55,56^ and age^57–61^.

We hypothesized that the coordination of inflammatory mediators early in the response dictates the subsequent trajectory. By studying propensity-matched groups of trauma patients with mild, moderate, or severe injury, we aimed to elucidate the mechanisms by which their corresponding inflammatory responses differed. Accordingly, we applied DyBN methodology to infer the inflammatory networks across injury severity. Our results showed that over a broad range of ISS, a core chemokine motif is observed consistently upstream of the central inflammation-associated cytokine IL-6. Based on DyBN, IL-6 appeared to receive more connections as severity of injury increased: for mild injury IL-6 only received input from MIG, whereas for moderate both MIG and MCP-1 contributed to the output and for severe MIG, MCP-1 and IP-10 contributed. In the absence of experimental perturbations to validate the model, we used trajectories of subgroups to show that the model can capture a range of dynamics that were observed in individual patients instead of only the mean behavior to which the model was calibrated.

Injury-induced inflammation has been linked to MODS, in-hospital outcomes, and longer-term morbidity^4–9^. Given this link between inflammation and trauma outcomes, and our hypothesis that the “chemokine switch” might represent a key determinant of self-sustaining vs. resolving inflammation, we sought to determine if the “chemokine switch” model could be linked in some way to proximal clinical outcomes. Analysis of the state transition diagrams of our Boolean model suggested that in the setting of moderate injury, initial conditions of the inflammatory mediators can determine how long it takes to reach the (resolving) steady state. In contrast, all trajectories reached steady state at the same time point under severe injury. This finding suggests that severity of injury may exert a greater impact than individual differences in baseline inflammatory mediators. Notably, when viewed as a pseudo-Kaplan–Meier curve, the state transition diagram of the “chemokine switch” Boolean model resembled the actual hospital discharge rate of moderately vs. severely injured patients. While this finding on its own does not prove a causal link between systemic inflammation and clinical outcomes as it does not consider the role of damage resolution or other factors affecting discharge, this result does suggest that the “chemokine switch” is related to other pathophysiological processes. Furthermore, this finding raises the possibility of patient-specific modeling and prediction of inflammation and clinical discharge based solely on the ISS (which is typically available within 24 h following hospital admission), along with initial circulating levels of MIG, MCP-1, IP-10, and IL-6. It is intriguing to speculate that this approach may also yield insights into the longer-term outcomes of critically ill patients.

There are multiple limitations to this study. The proposed logical model is not the only network that can give rise to the observed data and therefore does not represent a unique solution. Since the network is relatively small, a more rigorous effort to quantify model uncertainty can be made by permuting through all possible Boolean functions based on the network topology (i.e. combinations of AND/OR/NOT logic) and measuring the output of these models compared to the patient data. Alternatively, one may use Probabilistic Boolean Networks to determine the robustness of the model behavior to varying the logical functions encoding interactions^62^. Another limitation concerns the fact that our logical model is only quasi-mechanistic. The inferred interactions among the inflammatory mediators in our model are not reaction mechanisms but rather represent effects that involve activation and recruitment of cells as well as intracellular signaling and gene regulation leading to changes in expression/secretion of target cytokines. Another limitation is the possibility the assay kits used in this study detect differentially processed versions of the chemokines^63^ (e.g. the recently reported processed variant of IP-10, which has anti-inflammatory properties^64^), thereby accounting for differential pro- vs. anti-inflammatory effects in our model. We also note that although we are modeling both pro- and anti-inflammatory processes, we are not modeling healing explicitly.

We assume that injury is present throughout, and therefore affects the activation of the chemokines equally throughout the time course. However, IL-6 represents a potential bridge between pro- and anti-inflammatory responses^65^, and thus the inclusion of IL-6 as an output of the model could tie in to injury repair processes (e.g. repair pathways involving transforming growth factor-β1 ^66^). Notably, resolution of inflammatory mediators as determined by steady state in the model does not necessarily correspond to hospital discharge, as there are many other clinical factors besides inflammatory condition that determine discharge.

Lastly, the choice of synchronous updates is based on certain assumptions that may not hold true throughout the full time course under study. We assume that the events leading to the activation or suppression of one inflammatory mediator on another involve cell migration, signaling, and gene transcription, and therefore operate over relatively long timescales. This simplifying assumption leads to the further assumption that the interactions being modeled are similar across each chemokine-chemokine and chemokine-cytokine interaction. However, it is possible that these mediators interact on faster timescales. Indeed, there is evidence to suggest that the interaction among chemokines may be occurring at the level of competition for shared receptors, a relatively fast process^23,27,28^. In that case, some of the modeled interactions might necessitate a particular rank order of simulation updates or take into account stochastic effects (i.e. require asynchronous updates). Thus, a more thorough characterization of the biochemistry of interactions among cytokines could inform the appropriate choice of update scheme and also help refine the logical rules in the model.

There have been multiple calls for the integration of data-driven and mechanistic modeling as a means for overcoming the limited utility of pure machine learning approaches in biomedicine^67,68^. In support of this approach, our studies define a rational transition from data to data-driven models to mechanistic models in the context of a complex human disease and help decipher a novel mechanism for control of systemic acute inflammation. These insights combined with the logical modeling structure outlined herein may lead to novel diagnostic modalities, in which measurements of chemokines made early following admission may help prognosticate a given patient’s inflammatory and clinical trajectory.

## Methods

To define potentially novel control points in systemic acute inflammation induced by traumatic injury in humans, we employed a novel workflow to effectively integrate statistical and generative modeling (Fig. 1). We detail the relevant methods for this workflow below.

### Study approval

All human sampling was done following approval by the University of Pittsburgh Institutional Review Board (IRB; Protocol No. MOD08010232-19 / PRO08010232) and in accordance with the Declaration of Helsinki. Informed consent was obtained from each patient or next of kin as per IRB regulations. Patients eligible for enrollment in the study were at least 18 years of age, admitted to the ICU after being resuscitated, and per treating physician, were expected to live more than 24 h. Reasons for ineligibility were isolated head injury, pregnancy, and penetrating trauma. Laboratory results and other basic demographic data were recorded in the database via direct interface with the electronic medical record.

### Trauma patient enrollment and sampling

From a cohort of 472 blunt trauma survivors (330 males and 142 females, age 48.4 ± 0.9, ISS 19.6 ± 0.5), 48 mildly injured, 47 moderately injured, and 47 severely injured patients were matched using IBM SPSS Statistics case–control matching algorithm controlling for age and gender ratio (Table 1). Key clinical and inflammatory features of this patient cohort were reported recently^20,21^. Importantly, this sub-cohort represents the age, gender ratio, and mechan93ism of injury ratios of the general cohort. Serial blood samples were obtained from all patients (3 samples within the first 24 h and then from days 1 to 7 post-injury). The number and span of time points sampled for each patient varied, but all patients had at least three time points, all within the first 24 h post-injury.

### Analysis of inflammation biomarkers

The initial step in our workflow (Fig. 1A) involved obtaining a time-series dataset of circulating, protein-level inflammatory mediators. Blood samples were collected into citrated tubes via central venous or arterial catheters within 24 h of admission and daily up to 7 days post-injury. The blood samples were centrifuged, and plasma aliquots were stored in cryoprecipitate tubes at -80 °C for subsequent analysis of inflammatory mediators^20^. The human inflammatory MILLIPLEX MAP Human Cytokine/Chemokine Panel-Premixed 23-Plex (Millipore Corporation, Billerica, MA) and Luminex 100 IS (Luminex, Austin, TX) were used to measure plasma levels (in pg/ml) of interleukin (IL)-1β, IL-1 receptor antagonist (IL-1RA), IL-2, soluble IL-2 receptor-α (sIL-2Rα), IL-4, IL-5, IL-6, IL-7, IL-8 (CCL8), IL-10, IL-13, IL-15, IL-17, interferon (IFN)-γ, IFN-α2, IFN-γ inducible protein (IP)-10 (CXCL10), monokine induced by gamma interferon (MIG; CXCL9), macrophage inflammatory protein (MIP)-1α (CCL3), MIP-1β (CCL4), monocyte chemotactic protein (MCP)-1 (CCL2), granulocyte–macrophage colony stimulating factor (GM-CSF), Eotaxin (CCL11), and tumor necrosis factor alpha (TNF-α). The Luminex system was used in accordance to manufacturer’s instructions. NO_2_^-^/NO_3_^-^ was measured (in µM) using the nitrate reductase/Griess assay (Cayman Chemical Co., Ann Arbor, MI). The time courses of systemic inflammatory mediators for each patient sub-group are shown in Supplemental Figure S1. Two-Way Analysis of Variance (ANOVA) was carried out to analyze the changes in inflammatory mediators using SigmaPlot (Systat Software, San Jose, CA) as indicated.

### Dynamic Bayesian networks

The next phase of our workflow (Fig. 1B-D) involved inference of dynamic networks of circulating inflammatory mediators. Network inference using inflammatory mediator data was carried out in MATLAB (The MathWorks, Inc., Natick, MA), using a Dynamic Bayesian Network (DyBN) algorithm adapted from Grzegorczyk & Husmeier^69^ and recently used by our group^19,20,70,71^. Given time-series data, DyBN analysis provides a way of inferring causal relationships among variables (e.g. inflammatory mediators) based on probabilistic measure. Unlike standard correlative approaches, DyBNs consider the joint distribution of the entire dataset when making inferences about the dependencies between variables or nodes in the network. The values of each node are assumed to be distributed according to a chosen model (e.g. Gaussian) and the relationships among nodes are defined by the structure of the directed network and the corresponding conditional probability distributions of the interacting nodes. Network structure is inferred by a sampling technique that iteratively proposes candidate structures and evaluates them based on how well they fit the observed data using a specified scoring criterion, until reaching convergence on a network structure with the highest score. The algorithm uses an inhomogeneous dynamic changepoint model, with a Bayesian Gaussian with score equivalence (BGe) scoring criterion. The output of the aforementioned algorithm is a final graph structure indicating the interactions. This algorithm identified MCP-1, MIG, and IP-10 (Fig. 1B-D) as central nodes (i.e., nodes exhibiting feedback to themselves as well as bidirectional interactions among themselves), with IL-6 (Fig. 1E) as a common output node. These core interactions were therefore chosen as the basis of a quasi-mechanistic logical (Boolean) model (see below).

### Logical model

The final step in our workflow (Fig. 1E) was the generation of a quasi-mechanistic model derived from the inferred dynamic networks of systemic inflammation. To study the properties of the core chemokine network motif, the logical model initially consisted of only the chemokines MCP-1, MIG, and IP-10. This model was connected to injury severity as the initiating event, and IL-6 was added as a key output cytokine, as inferred from DyBN inference (see above; Fig. 1E). The predicted trajectories of these variables were compared to inflammatory dynamics observed in sub-groups of trauma patients, stratified based on injury severity. The elements in the model were connected as inferred in the DyBN analyses carried out on patients with mild, moderate, or severe trauma (Fig. 1B-D). Edges were assigned as stimulating or inhibiting based on plausible mechanisms upon reviewing the literature and fine-tuned to reproduce observed cytokine trajectories. Logic rules that defined the combination of multiple inputs were chosen in a similar fashion. We started all elements as strictly two-state Boolean variables, except injury severity, which needed to have three states to represent mild, moderate, and severe injury. However, in order to reproduce the clinical data and avoid spurious oscillations, MCP-1, IP-10, and IL-6 were modified to have three states as well. Three-state elements were encoded by splitting their corresponding variables to “high” and “low” variables, the sum of which gives the final state for that element. Model simulations were run with synchronous updates and fixed injury severity, but with random initial conditions for all other variables, in order to mimic the variability of initial cytokine and chemokine values observed in the patient population. Since the initial states were specified as “random”, we ran 1000 simulations to ensure that we covered all possible permutations of initial states. Results are presented as mean and standard deviation of the 1000 simulations. The model was encoded and run using Booleannet software^72^ and can be found on GitHub (https://github.com/nazhar/ChemokineSwitch).

### Statistical analysis

All data were analyzed using SigmaPlot 11 software (Systat Software, Inc., San Jose, CA). Statistical comparisons were performed using either Kruskal–Wallis one-way analysis of variance (ANOVA) followed by the Dunn’s post hoc test (for continuous data) or Fisher's exact test (for categorical data), as appropriate.

## Supplementary Information

Supplementary Information 1.

Supplementary Information 2.

## Data Availability

All data generated or analyzed during this study are included in this published article (and its Supplementary Information files).
