# Supplementary material for: A putative “chemokine switch” that regulates systemic acute inflammation in humans
Source: Sci Rep. 2021 May 6;11:9703. doi: 10.1038/s41598-021-88936-8 (PMC8102583; doi:10.1038/s41598-021-88936-8)
Supplement: Supplementary file 1 — Supplementary Information 1. [file 41598_2021_88936_MOESM1_ESM.pdf]

A Putative "Chemokine Switch" that Regulates Systemic Acute Inflammation in Humans

Nabil Azhar[1,2,3], Rami A. Namas[1,2], Khalid Almahmoud[1], Akram Zaaqoq[1], Othman A. Malak[1], Derek Barclay[1], Jinling Yin[1], Fayten El-Dehaibi[1], Andrew Abboud[1], Richard L. Simmons[1], Ruben Zamora[1,3], Timothy R. Billiar[1], Yoram Vodovotz[1,3,4,*]

[1]Department of Surgery, University of Pittsburgh, Pittsburgh, PA 15213
[2]Department of Computational and Systems Biology, University of Pittsburgh, Pittsburgh, PA 15213
[3]Center for Inflammation and Regeneration Modeling, McGowan Institute for Regenerative Medicine, University of Pittsburgh, Pittsburgh, PA 15219
[4]Center for Systems Immunology, University of Pittsburgh, Pittsburgh, PA 15213

[*]**Correspondence**
Yoram Vodovotz, PhD.
Department of Surgery
University of Pittsburgh
W944 Starzl Biomedical Sciences Tower
200 Lothrop St.
Pittsburgh, PA 15213
Tel.: 412-585-0115
E-mail: vodovotzy@upmc.edu

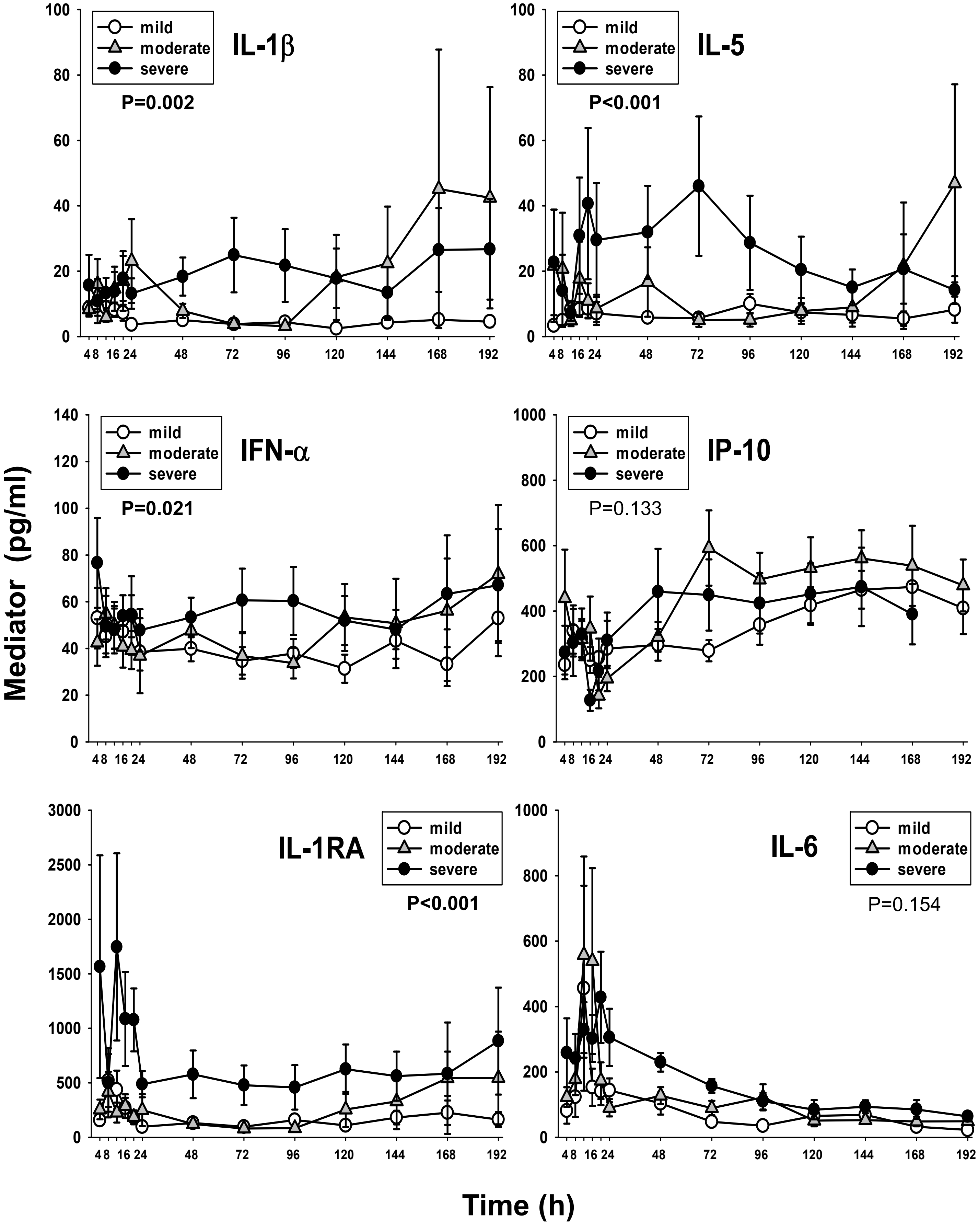
IL-1β
P=0.002
IL-5
P<0.001
IFN-α
P=0.021
IP-10
P=0.133
IL-1RA
P<0.001
IL-6
P=0.154
mild
moderate
severe
Mediator (pg/ml)
Time (h)
4 8 16 24 48 72 96 120 144 168 192

Fig. S1

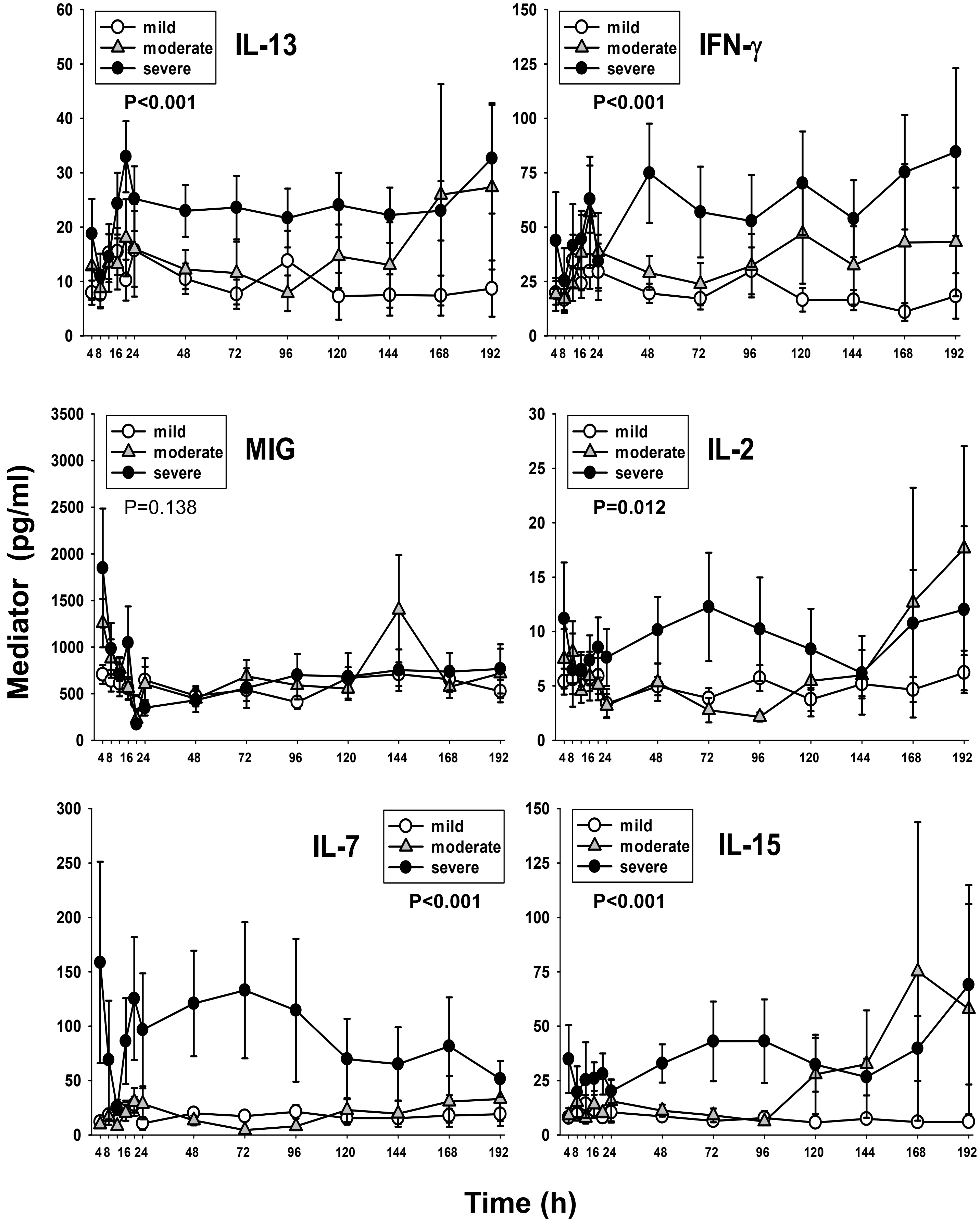
IL-13
P<0.001
IFN-γ
P<0.001
MIG
P=0.138
IL-2
P=0.012
IL-7
P<0.001
IL-15
P<0.001
mild
moderate
severe
Mediator (pg/ml)
Time (h)
4 8 16 24 48 72 96 120 144 168 192

Fig. S1

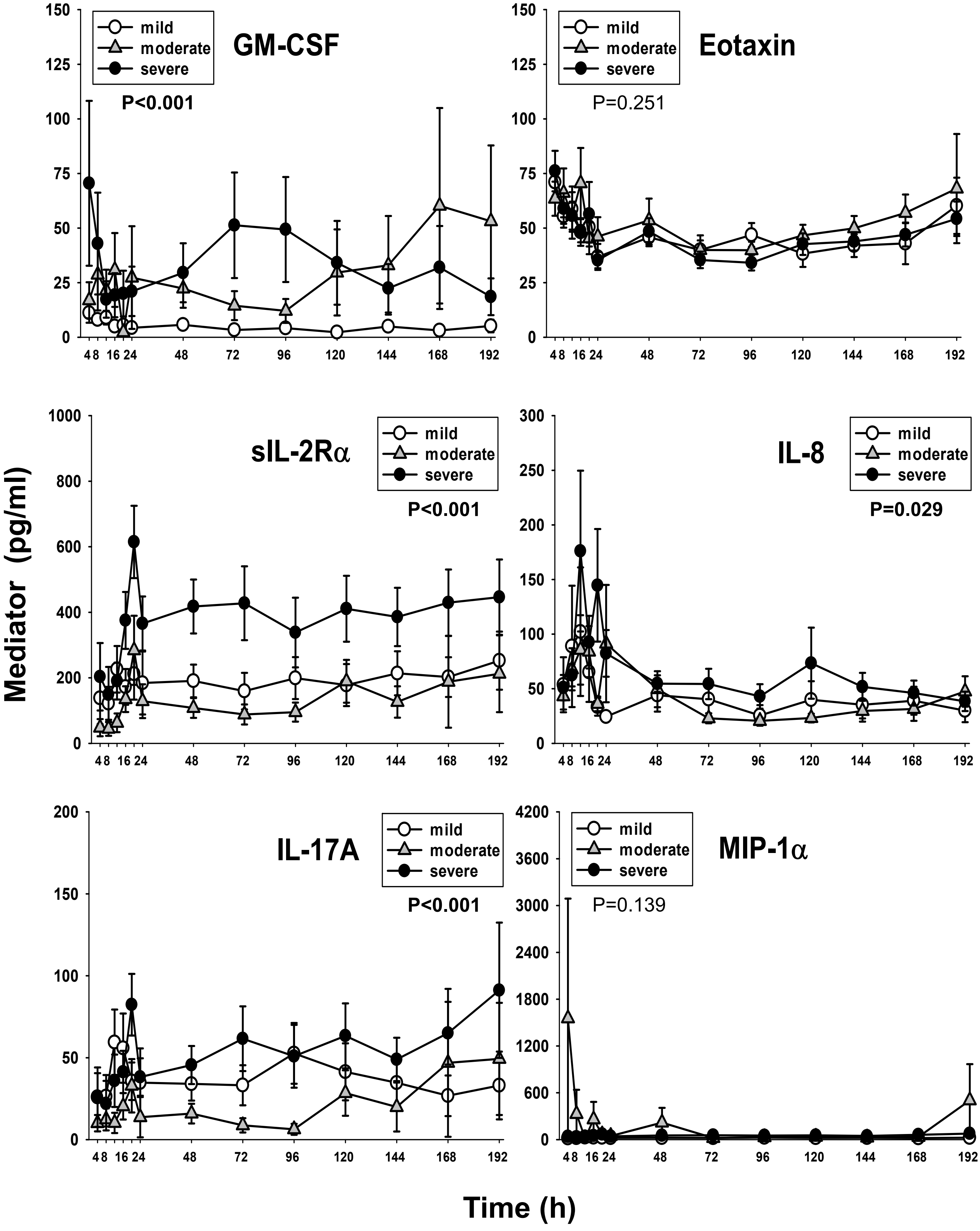

GM-CSF
P<0.001
Eotaxin
P=0.251
sIL-2Rα
P<0.001
IL-8
P=0.029
IL-17A
P<0.001
MIP-1α
P=0.139
mild
moderate
severe
Mediator (pg/ml)
Time (h)

Fig. S1

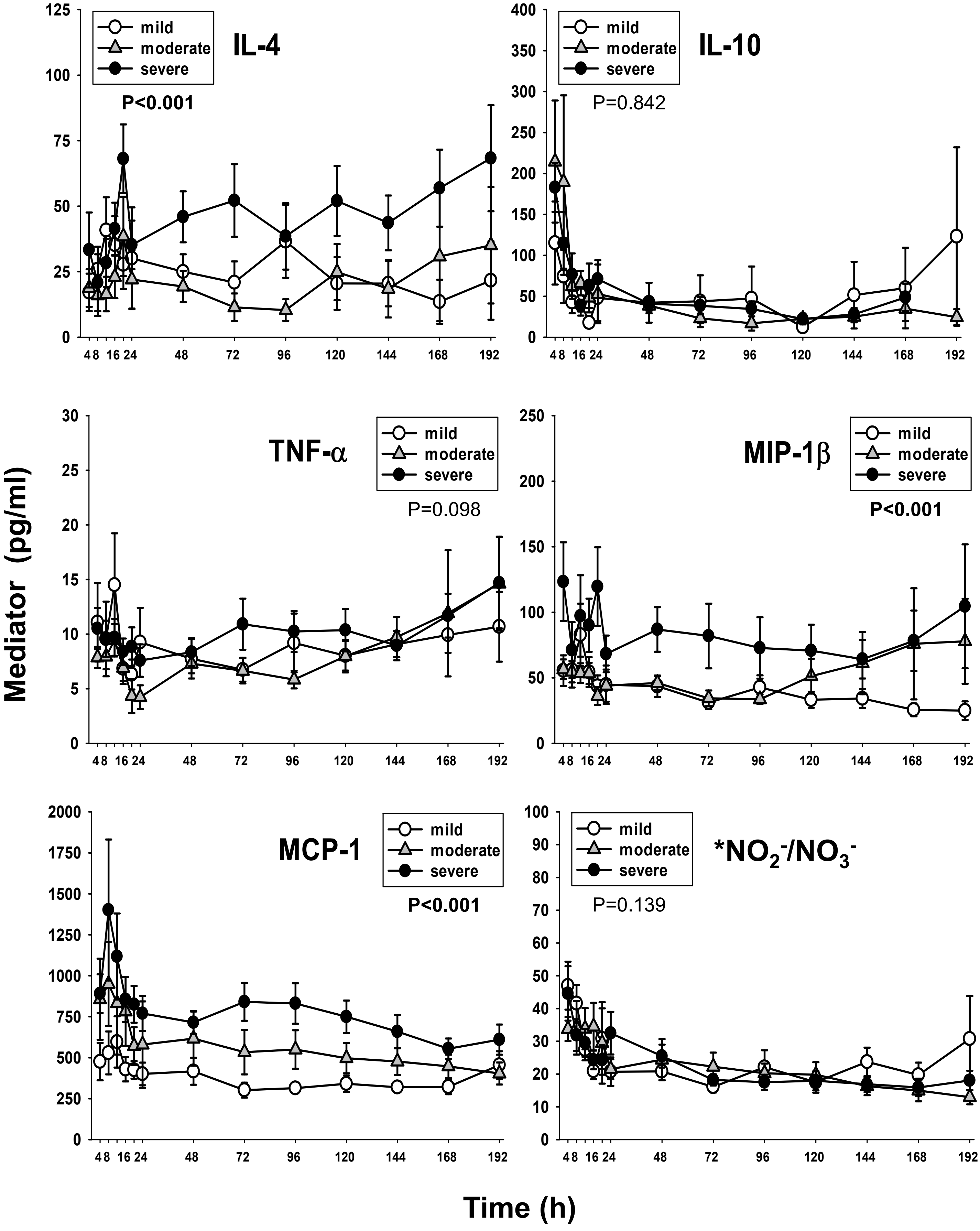
IL-4
P<0.001
IL-10
P=0.842
TNF-α
P=0.098
MIP-1β
P<0.001
MCP-1
P<0.001
*NO_2^-/NO_3^-
P=0.139
mild
moderate
severe
Mediator (pg/ml)
Time (h)
0
25
50
75
100
125
150
200
250
300
350
400
5
10
15
20
30
1000
1250
1500
1750
2000
500
750
40
60
70
80
90
4
8
16
24
48
72
96
120
144
168
192

Fig. S2

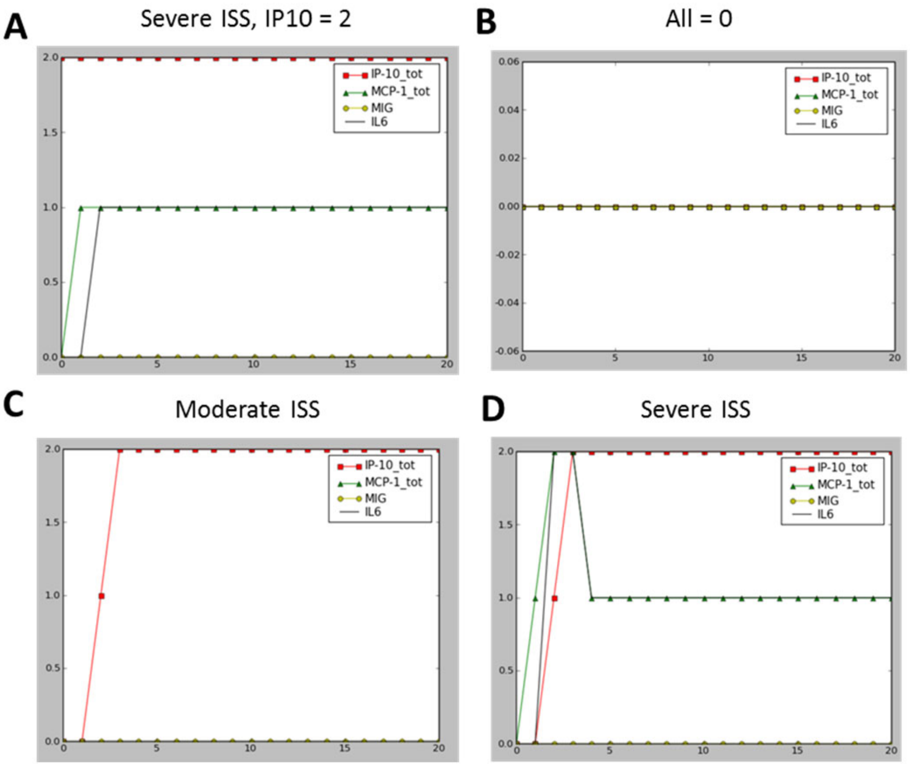

Fig. S2. General Boolean model behavior for different baseline initial conditions. *Panel A*: Severe injury and all mediators start at 0 except IP-10 initial value set to 2 (high). *Panel B*: All mediators and injury initial value set to 0. *Panel C*: Moderate injury and all mediators start at 0. *Panel D*: Severe injury and all mediators start at 0.

Fig. S3

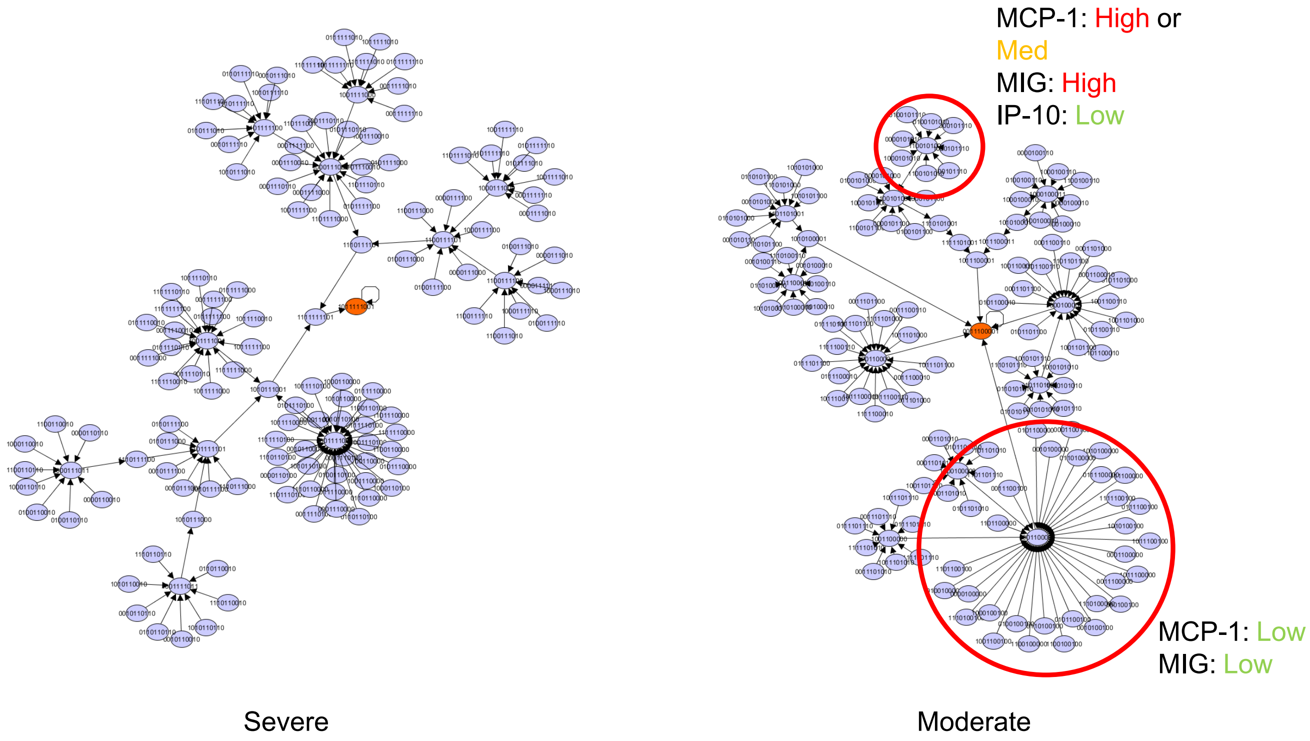

Fig. S3. State transition graphs for Severe and Moderate Injury simulations. Each node represents a snapshot of the current levels for all mediators. Arrows indicate trajectory of simulation from one time-step to the next. Outermost nodes are the initial starting states and node with self-directed arrow is the final steady state (i.e. the model has only one attractor, highlighted in orange)

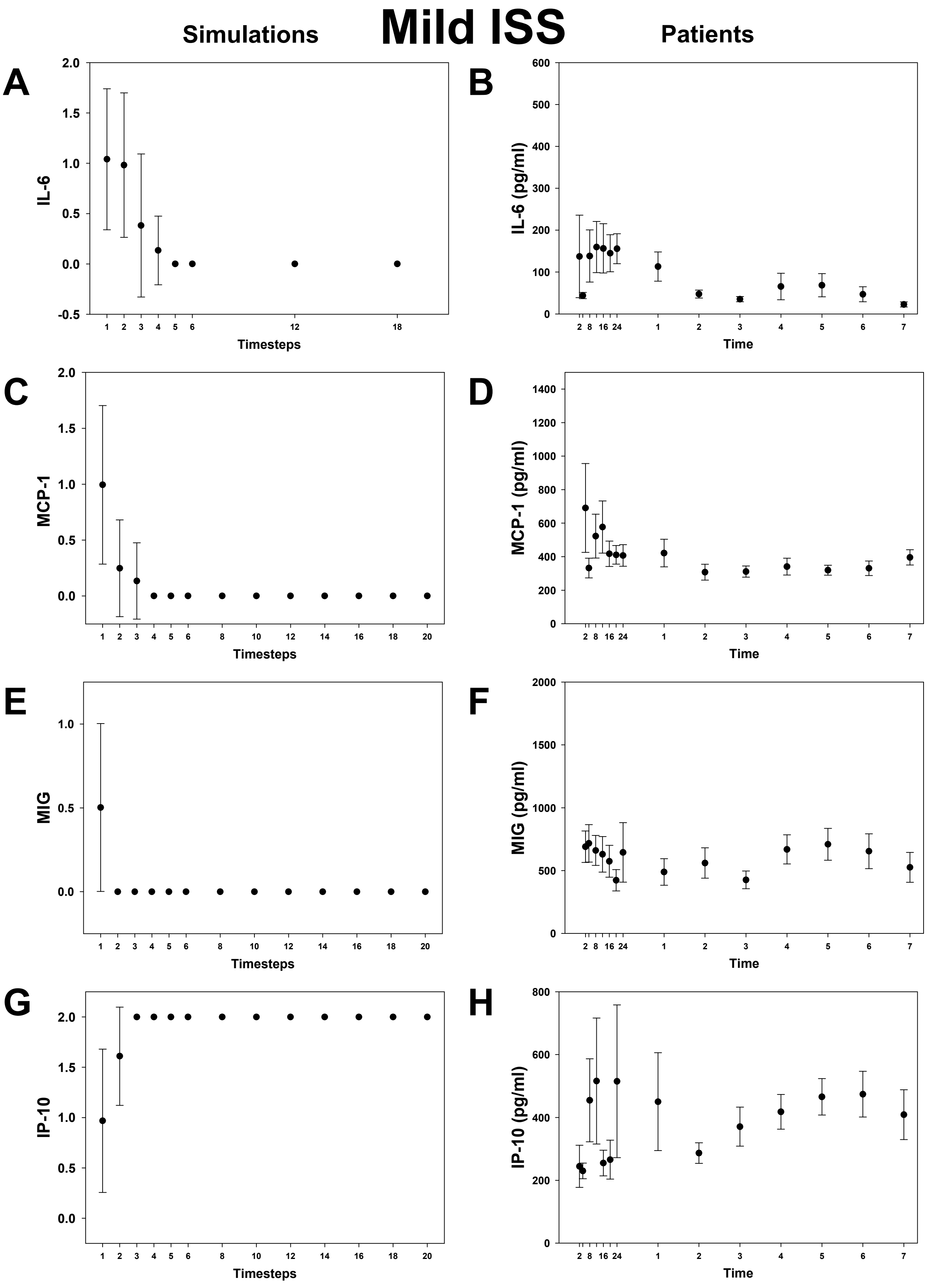

Fig. S4. Inflammatory mediator trajectories for Mild Injury: Simulations vs. data from trauma patients. *Left column*: 500 simulations were run with random initial conditions. Plot shows mean plus standard error for each time step. *Right column*: Patient data shown as mean with standard error for each time point.

Table S1. Rules for Logical Model. Update rules for each node in the logical model. "X* =" indicates the value of mediator X at the current time point is computed by evaluating the expression on the right hand side of the "=" symbol.

**IP10_hi* = IP10 or not (MCP1 or MIG)**
**IP10* = not ISS or not (MCP1 and MIG)**
**MCP1* = ISS_hi or (MCP1 and not IP10_hi)**
**MCP1_hi* = ISS_hi and MCP1 and not IP10_hi**
**MIG* = ISS and MIG and not IP10_hi and not MCP1**
**IL6* = MIG or MCP1**
**IL6_hi* = MCP1 and not IP10_hi**
