# Supplementary material for: A putative “chemokine switch” that regulates systemic acute inflammation in humans
Source: Sci Rep. 2021 May 6;11:9703. doi: 10.1038/s41598-021-88936-8 (PMC8102583; doi:10.1038/s41598-021-88936-8)
Supplement: Supplementary file 2 — Supplementary Information 2. [file 41598_2021_88936_MOESM2_ESM.docx]

**Supplementary Materials**

Figure S1. Time courses of systemic inflammatory mediators. Inflammatory mediators were assayed for each patient sub-group analyzed by Two-Way Analysis of Variance (ANOVA) as described in *Materials and Methods*. P values are indicated in each panel (in bold for all P < 0.05).

Figure S2. General Boolean model behavior for different baseline initial conditions. *Panel A*: Severe injury and all mediators start at 0 except IP-10 initial value set to 2 (high). *Panel B*: All mediators and injury initial value set to 0. *Panel C*: Moderate injury and all mediators start at 0. *Panel D*: Severe injury and all mediators start at 0.

**Figure S3. State transition graphs for Severe and Moderate Injury simulations.** Each node represents a snapshot of the current levels for all mediators. Arrows indicate trajectory of simulation from one time-step to the next. Outermost nodes are the initial starting states and node with self-directed arrow is the final steady state (i.e. the model has only one attractor, highlighted in orange)

**Fig. S4. Inflammatory mediator trajectories for Mild Injury: Simulations vs. data from trauma patients.** *Left column:* 500 simulations were run with random initial conditions. Plot shows mean plus standard error for each time step. *Right column:* Patient data shown as mean with standard error for each time point.

**Table S1****. Rules for Logical Model.** Update rules for each node in the logical model. “X* =” indicates the value of mediator X at the current time point is computed by evaluating the expression on the right-hand side of the “=” symbol.
